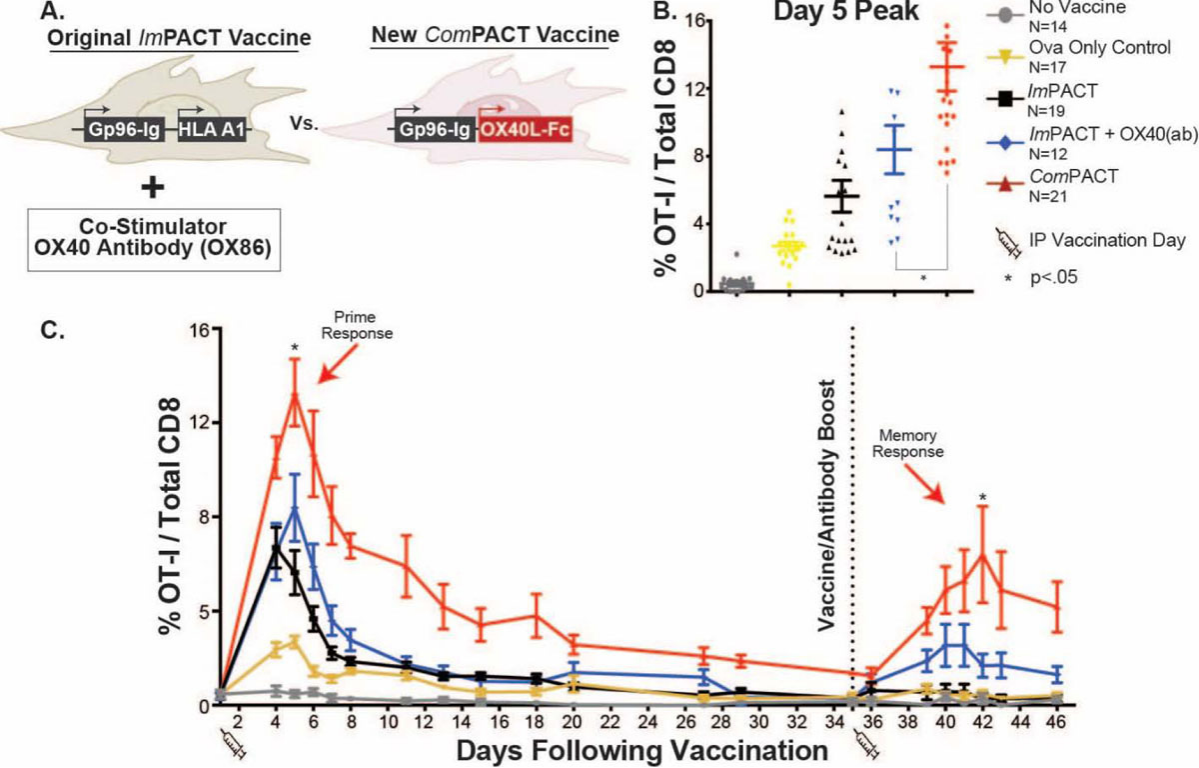# Locally secreted Fc-OX40L is superior to systemic, antibody mediated, OX40 costimulation for combination immunotherapy

**DOI:** 10.1186/2051-1426-3-S2-P371

**Published:** 2015-11-04

**Authors:** George Fromm, Jason Rose, Suresh Desilva, Taylor Schreiber

**Affiliations:** 1 Heat Biologics Inc., Durham, NC, USA

## 

The dramatic clinical success of checkpoint inhibitory therapy (anti-CTLA-4 and anti-PD-1) in a small percentage of patients has highlighted the need to identify combination approaches that may increase the frequency of responders. Two immunotherapy modalities that are proposed to synergize both with each other, and with checkpoint inhibitors are therapeutic vaccines and T cell co-stimulators. To prioritize which T cell co-stimulators enhance the efficacy of an allogeneic, gp96-Ig secreting, cell-based vaccine, we investigated the activity of agonistic antibodies targeting OX40, 4-1BB and ICOS administered together with gp96-Ig vaccines (*ImPACT*). These data demonstrated that antigen-specific CD8+ T cell expansion is significantly enhanced by OX40, but not 4-1BB or ICOS stimulation. Because T cell co-stimulation occurs at the site of immunization, we then asked when co-expression of Fc-OX40L by the gp96-Ig secreting allogeneic vaccine cells (*ComPACT*) would provide comparable co-stimulation to systemically administered OX40 agonist antibodies (Figure A). Interestingly, these data demonstrated that locally secreted Fc-OX40L provided superior priming of antigen-specific CD8+ T cells (peak of 14.05% of total CD8+) as compared to combinations with OX40 antibodies (8.9%) or vaccine alone (5.2%) (Figure B). The reason for the beneficial response was related to more potent activation of CD127^+^KLRG-1^-^ memory precursor cells by the Fc-OX40L expressing vaccine. Importantly, antigen-specific CD8+ cells stimulated by vector-encoded Fc-OX40L demonstrated a protracted contraction phase after priming, and remained at high levels in both tissues and peripheral blood for 3 weeks after priming (Figure C). Systemic administration of OX40 antibodies also led to proliferation of antigen non-specific CD4+ T cells, Treg and systemic increases in IL-4, IL-5, IL-6, TNFα and IFNα. Importantly, vaccine-expressed Fc-OX40L led to high frequencies of IFNα^+^, TNFα^+^, granzyme-b^+^ and IL-2^+^ antigen-specific CD8+ T cells at both priming and boosting, which enhanced rejection of established CT26 tumors. These data demonstrate that vaccination and co-stimulation can be approached with a single cell-based product, and that locally delivery of a vaccine and T cell co-stimulator enhances the primary and memory responses of highly activated CD8+ T cells as compared to co-stimulatory antibodies. This may lead to more rapid development of combination immunotherapeutics with reduced toxicity, as a result of increased specificity, and a simpler cost structure than combining multiple independent biologic medicines.

**Figure 1:**